# Analytical and Numerical Investigations into Hemisphere-Shaped Electrostatic Sensors

**DOI:** 10.3390/s140814021

**Published:** 2014-07-31

**Authors:** Jun Lin, Zhong-Sheng Chen, Zheng Hu, Yong-Min Yang, Xin Tang

**Affiliations:** Science and Technology on Integrated Logistics Support Laboratory, National University of Defense Technology, Changsha 410073, China; E-Mails: czs_study@sina.com (Z.-S.C.); zhenghu@nudt.edu.cn (Z.H.); yangyongmin@163.com (Y.-M.Y.); tangxin11_nudt@163.com (X.T.)

**Keywords:** electrostatic monitoring, hemisphere-shaped sensors, Green function, spatial sensitivity, induced charge

## Abstract

Electrostatic sensors have been widely used in many applications due to their advantages of low cost and robustness. Their spatial sensitivity and time-frequency characteristics are two important performance parameters. In this paper, an analytical model of the induced charge on a novel hemisphere-shaped electrostatic sensor was presented to investigate its accurate sensing characteristics. Firstly a Poisson model was built for electric fields produced by charged particles. Then the spatial sensitivity and time-frequency response functions were directly derived by the Green function. Finally, numerical calculations were done to validate the theoretical results. The results demonstrate that the hemisphere-shaped sensors have highly 3D-symmetrical spatial sensitivity expressed in terms of elementary function, and the spatial sensitivity is higher and less homogeneous near the hemispherical surface and *vice versa*. Additionally, the whole monitoring system, consisting of an electrostatic probe and a signal conditioner circuit, acts as a band-pass filter. The time-frequency characteristics depend strongly on the spatial position and velocity of the charged particle, the radius of the probe as well as the equivalent resistance and capacitance of the circuit.

## 1. Introduction

In recent years, due to their advantages of low cost and robustness, various electrostatic sensors have been widely used for detecting abnormal debris present in the gas path of the engine from faults [1,2] or measuring particle concentration and size in gas-solid flows [3–24]. At present, intrusive sensors and non-intrusive sensors are the main categories.

In practice, sensing characteristics are very important for electrostatic sensors. Optimal sensor designs are the key to obtaining better sensing characteristics. In order to provide guidelines for sensor design, two key points are important. First, intrusive sensors have high spatial sensitivity, but they affect the gas flow. This contradiction raises difficulties in the sensor design process. Generally, a trade-off needs to be made between the spatial sensitivity and the gas flow disturbance. In order to achieve that, accurate sensor models have to be built. Second, while using these models to make optimal designs, analytical solutions are always difficult to obtain because of their irregular Dirichlet boundaries. Therefore, current designs of rod-shaped sensors [4] and intrusive round-head-screw-shaped sensors [5] always use Finite element modeling (FEM) methods instead of obtaining analytical solutions to study sensing characteristics. However, the corresponding calculation time of FEM methods is usually very long.

In some cases, such as gas path monitoring, intrusive sensors are not allowed because they affect the gas flow. Conversely, non-intrusive sensors are widely used, since they cause no disturbance to the gas flow, but most of them have little spatial sensitivity due to the electrostatic pipeline shields or their intrinsic sensing characteristics. Such sensors include thin-plate-shaped probes [6–8], square-shaped probes [9,10], ring-shaped probes [11–23], small probes with arbitrary shape [24], and so on. Ghazali *et al.* [6] and Peng *et al.* [10] built mathematical models of thin-plate-shaped sensors and square-shaped sensors, respectively. However, the analytical functions are so complicated that they have to be calculated indirectly by an approximate numerical calculation. Vata Zhin *et al.* [24] built by Green function a theoretical and laboratory model for gas path monitoring. In this model, an arbitrary probe was located outside the jet, and the probe dimension was supposed small enough to not change the induced electric field outside the jet. However, these assumptions are invalid in practice. Induced signals are weak due to the electrostatic pipeline shields. Numerical models of ring-shaped sensors and sensors array were built based on FEM methods in [15,20–23]. However, as mentioned before, FEM methods are approximate and usually will take a long time to build the model. Furthermore, it is difficult to detect localized flow regimes with ring-shaped sensors because the induced signals are from all particles in the cross-section of the pipeline. Also they are usually expensive and difficult to install due to the fact they take the form of a spool piece installed in line with pipework [4]. Unlike those two types of sensors, in this paper, a hemisphere-shaped sensor was proposed. Since the probe size is much smaller than the radius of the pipeline, it causes fewer disturbances to the gas flow. It also has 3D-symmetrical and concise spatial sensitivity functions, so that the corresponding calculation in signal process methods becomes quite easy. These advantages make it be much promising in many applications, such as an electrical charge tomography system [8].

To date, few works have been done on hemisphere-shaped sensors. In order to study their sensing characteristics and provide guidelines for sensors design, analytical and elementary mathematical functions of hemisphere-shaped sensors are formulated for the first time in this paper, and then the corresponding numerical calculations were done to verify the theoretical results. The main contributions of this paper include two aspects: the first innovation is to derive the mathematical functions of hemisphere-shaped sensors. This way, it should be easier, faster and more accurate to study their sensing characteristics than using FEM methods, since these functions are concise and 3D-symmetrical. The second is that more parameters were taken into account to completely investigate the performance of sensors, such as the spatial position and velocity of the charged particle, the radius of the probe and so on. Especially, the equivalent resistance and capacitance of the whole signal processed circuit are discussed in detail in this paper, which can provide better guidelines for engineering applications. In fact, these factors have important effects on the time-frequency characteristic of sensors, but they were often neglected or forgotten in many papers [6,8,16,20].

The outline of this paper is summarized as follows: first, the Poisson model was built for electric fields produced by charged particles. Then the elementary spatial sensitivity and time-frequency response functions of hemisphere-shaped sensors were derived by a Green function. Finally, the numerical calculations performed to validate the theoretical results are presented and discussed.

## 2. Mathematical Model

### 2.1. Configuration of Hemisphere-Shaped Sensors

**Figure 1.**
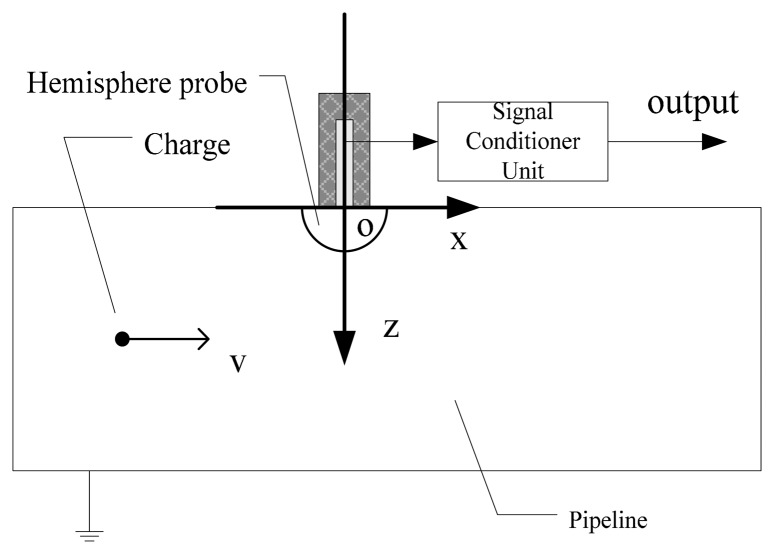
Schematic of the hemisphere-shaped sensor installation.

As shown in Figure 1, the hemisphere-shaped sensor installation comprises a hemisphere probe and a signal conditioner unit. For gas path monitoring, the probe detects electrostatic charges present within the pipeline due to exhaust gas and debris. It was manufactured from a nickel alloy to meet the high temperature requirements of the engine. The signal conditioner unit receives a charge signal from the probe and converts it to a voltage signal suitable for processing and acquisition. For the current application, an amplifier and a filter were built into the signal conditioner unit.

### 2.2. The Poisson Model of Electric Fields

The motion of a charged particle is essentially the nonstationary one consistent with the motion of the gas flow. Therefore a changed electric field appears inside the pipeline, which is determined by the charged particle motion. It can be seen from Figure 1, the center of the hemisphere probe is first set as the origin of the Descartes coordinate system. Then the axial orientation of the pipeline, the axial and vertical orientations of the hemisphere probe are set as the ***x***-axis, ***z***-axis and ***y***-axis of the global coordinate system, respectively. The arbitrary point in the pipeline can be denoted as *P*(*x*, *y*, *z*). In the considered approximation, it is assumed that volume charge density *ρ*(*P*, *t*) is a prescribed function and simulates different charge motions in the pipeline. Here *t* is the time. Since the inducted time is insignificant (10^−19^ s), the interaction between the probe and moving charge particles could be treated as a pure electrostatic field. Hence the theoretical model of the hemisphere-shaped sensor is determined by the Poisson equation and Dirichlet boundary conditions as follows:

(1) $$\Delta \varphi (P,t)=-\frac{\rho (P,t)}{\varepsilon },s.t.\{\begin{array}{ll} \varphi (P)=0 & P\in S_{P}\\ \varphi (P)=\phi (\text{t}) & P\in S_{N} \end{array}$$

where *ϕ*(*P*) is the electric potential, *ε* is the dielectric permittivity of free space. *S_p_*, *S_N_* are the outer boundaries of the pipeline and the probe. The conducting probe forms an equipotential volume. The potential of the probe at time *t* is denoted as *ϕ*(*t*). Since Equation (1) describes an external Dirichlet boundary problem, the hidden boundary condition is $\underset{P\to \infty }{\lim}\varphi (P)=0$. As shown in [25], the solution to this problem is unique. The solution of an arbitrary Poisson Equation can be written by Green function as follows:

(2) $$\varphi (P,t)=\int_{v}G(P,P_{0})\rho (P_{0},t)dP_{0}-\varepsilon \oint_{\Gamma }f(P_{0})\frac{\partial G(P,P_{0})}{\partial {\text{n}}_{P_{0}}}dS_{P_{0}}$$

Here Γ is the outer boundary of the region, *f* is the potential on the boundary. Thus Green function holds based on Equation (1):

(3) $$\Delta G(P,P_{0})=-\delta (P,P_{0})/\varepsilon ,s.t.G{\left(P,P_{0}\right)}_{|p\in \Gamma }=0$$

**Figure 2.**
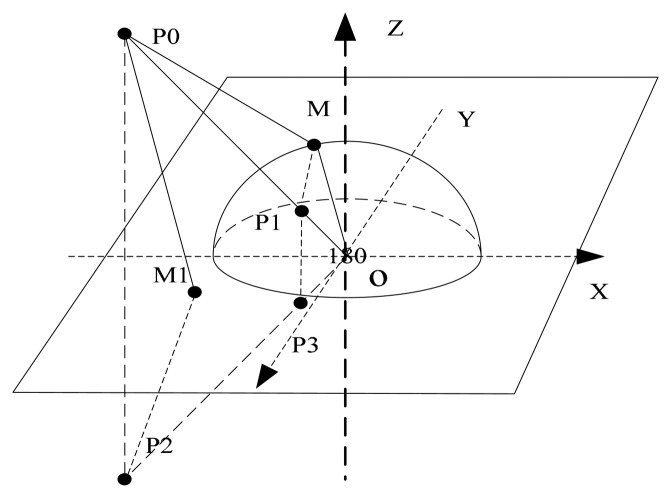
The electric field distribution of the hemisphere probe.

The Green function in Equation (3) describes the potential field distribution inside the pipeline when the unit charge *δ*(*P*, *P*_0_) is located at P0(*x*_0_, *y*_0_, *z*_0_) and the probe is grounded. When a point charge is outside the grounded probe, the Green function can be resolved by the image charges method. The potential inside the pipeline is affected by the grounded boundary of the pipeline. If the radius of the pipeline is much longer than the radius of the probe, the electric field on the boundary of the pipeline is similar to one on the infinite plane at the same position. The calculated results in [10,11] can validate the consequence. Thus the electric field near the hemisphere probe is shown in Figure 2.

It is assumed that the radius of the hemisphere probe is *a*, point *M* is on the hemispherical surface and *M*_1_ is on the plane *z* = 0. A positive point charge +*q*_0_ is located at *P*_0_(*x*_0_, *y*_0_, *z*_0_). The potential on the hemispherical surface and plane *z* = 0 is zero, which can be denoted respectively as *φ*(*M*) = 0, *φ*(*M*_1_). First, the Equation *φ* (*M*) = 0 is considered. An image charge *q*_1_ is located at *P*_1_ on the line connecting the origin and *q*_0_. Selecting the suitable location and magnitude of the image charge, the electric field due to the charge *q*_0_ outside the grounded conducting hemispherical surface can be created by the charge *q*_0_ and *q*_1_. Then the electric field at M can be formulated as follows:

(4) $$\varphi (M)=\frac{q_{0}}{4\pi \varepsilon \left|MP_{0}\right|}+\frac{q_{1}}{4\pi \varepsilon \left|MP_{1}\right|}=0$$

Choosing *P*_1_ to satisfy the condition Δ*OP*_1_*M* ∼ Δ*OMP*_0_, one will have:

(5) $$\frac{\left|OP_{1}\right|}{\left|OM\right|}=\frac{\left|OM\right|}{\left|OP_{0}\right|}=\frac{\left|MP_{1}\right|}{\left|MP_{0}\right|}=-\frac{q_{1}}{q_{0}}$$

Substituting Equations $|OP_{0}|=b=\sqrt{x_{0}^{2}+y_{0}^{2}+z_{0}^{2}}$ and |*OM*| = *a* into Equation (5), then the magnitude of the image charge is $q_{1}=-\frac{aq_{0}}{b}$, at $P_{1}(\frac{a^{2}}{b^{2}}(x_{0},y_{0},z_{0}))$. To meet the condition *φ*(*M*_1_) = 0, as shown in Figure 2, image charges −*q*_2_ = *q*_0_, $q_{3}=\frac{aq_{0}}{b}$ are symmetrically placed at *P*_2_(*x*_0_, *y*_0_, −*z*_0_), $P_{3}(\frac{a^{2}}{b^{2}}(x_{0},y_{0},-z_{0}))$ on the other side of the plane *z* = 0, respectively. Hence one will have |*M*_1_*P*_0_| = |*M*_1_*P*_2_|, |*M*_1_*P*_1_| = |*M*_1_*P*_3_| and the boundary conditions are satisfied as follows:

(6) $$\begin{array}{ll} \varphi (M_{1})= & \frac{1}{4\pi \varepsilon }(\frac{q_{0}}{\left|M_{1}P_{0}\right|}+\frac{q_{1}}{\left|M_{1}P_{1}\right|}+\frac{q_{2}}{\left|M_{1}P_{2}\right|}+\frac{q_{3}}{\left|M_{1}P_{3}\right|})=0\\ \varphi (M)= & \frac{1}{4\pi \varepsilon }(\frac{q_{0}}{\left|MP_{0}\right|}+\frac{q_{1}}{\left|MP_{1}\right|}+\frac{q_{2}}{\left|MP_{2}\right|}+\frac{q_{3}}{\left|MP_{3}\right|})=0 \end{array}$$

Since the image charges are located outside the pipeline region, the unique Green function can be written as:

(7) $$\begin{array}{l} G(P,P_{0})=\frac{1}{4\pi \varepsilon }(\frac{1}{\left|PP_{0}\right|}-\frac{a}{b\left|PP_{1}\right|}-\frac{1}{\left|PP_{2}\right|}+\frac{a}{b\left|PP_{3}\right|})\\ \left|PP_{0}\right|,\left|PP_{2}\right|=\sqrt{{\left(x-x_{0}\right)}^{2}+{\left(y-y_{0}\right)}^{2}+{\left(z\mp z_{0}\right)}^{2}}\\ \left|PP_{1}\right|,\left|PP_{3}\right|=\sqrt{{\left(x-\frac{a^{2}}{b^{2}}x_{0}\right)}^{2}+{\left(y-\frac{a^{2}}{b^{2}}y_{0}\right)}^{2}+{\left(z\mp \frac{a^{2}}{b^{2}}z_{0}\right)}^{2}} \end{array}$$

### 2.3. The Induced Charge of the Hemisphere Probe

The surface charge density *σ* on the hemisphere probe is:

(8) $$\sigma =\varepsilon E_{\text{n}}=-\varepsilon \frac{\partial \varphi }{\partial \text{n}}$$

where *E*_n_ is the electrostatic field on the surface of the probe, **n** is the normal vector. When the Equation *q*_0_ = 1 is set, the induced charge *Q* can be calculated as follows based on Equations (2) and (8):

(9) $$Q=\oint_{\Gamma }\sigma ds=Q_{1}+Q_{2}$$

Here $Q_{1}=-\varepsilon \oint_{\Gamma }\frac{\partial G(P,P_{0})}{\partial \text{n}}ds_{P}$, *Q*_2_ = *λϕ*(*t*), $\lambda =\varepsilon^{2}\oint_{\Gamma }\frac{\oint_{\Gamma }\frac{\partial G(P,P_{0})}{\partial \text{n}}ds_{P}}{\partial {\text{n}}_{P_{0}}}ds_{P_{0}}$. Γ is the surface of the hemisphere probe. The physical explanation of *λ* is the capacitance of the probe itself, which plays an important role in the system response. Based on Gauss' law, Equation (10) is given:

(10) $$\begin{array}{l} Q_{1}=-\frac{a}{b}=-\frac{a}{\sqrt{x_{0}^{2}+y_{0}^{2}+z_{0}^{2}}}\\ \lambda =-\varepsilon \oint_{\Gamma }\frac{Q_{1}(P_{0})}{\partial {\text{n}}_{p_{0}}}ds_{p_{0}}=2\pi a\varepsilon \end{array}$$

The total induced charges of the hemisphere probe comprise *Q*_1_ and *Q*_2_. *Q*_2_ is proportional to the potential of the hemisphere probe, which reflects the accumulated charges due to the capacitance of the probe itself. *Q*_1_ is determined by the distribution of charges within the pipeline. It is not convenient to calculate *Q* by Green function directly when *ρ*(*P*, *t*) is prescribed. According to previous studies, the spatial sensitivity of the hemisphere probe is defined as the absolute value of the induced charge on the probe from a unity point charge in its sensing zone [16]. It can be expressed as:

(11) $$S(x,y,z)=\left|Q_{1}\right|=\frac{a}{\sqrt{x^{2}+y^{2}+z^{2}}}$$

The total charges of the hemisphere probe can be written as follows:

(12) $$Q(t)=-\int_{v}S(P)\rho (P,t)dP+2\pi a\varepsilon \phi (t)$$

Obviously, the spatial sensitivity function is elementary. Using this function, it is easier and faster to study sensing characteristics of hemisphere-shaped sensors with the advantages of accuracy and less complexity. Furthermore, the spatial sensitivity is 3D-symmetrical, which evidently has more promise in many applications.

### 2.4. The Output Signal of the Hemisphere-Shaped Sensor

**Figure 3.**
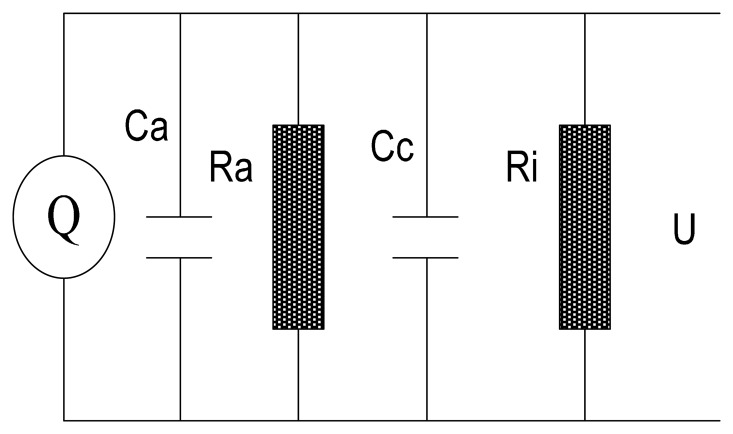
The equivalent circuit.

The signal conditioner unit converts a charge signal to a voltage signal suitable for processing and acquisition. The equivalent circuit of the signal conditioner is shown in Figure 3. It comprises an equivalent current source, a grounded capacitance *C_a_*, a grounded resistance *R_a_*, a cable capacitance *C_c_* and an amplifier resistance *R_i_*. Therefore, $R=\frac{R_{a}R_{i}}{R_{a}+R_{i}}$, *C* = *C_a_* + *C_c_* are the equivalent resistance and capacitance of the whole circuit, respectively. Assuming the initialization of the circuit is zero, then the Fourier transform of the circuit equation can be obtained as follows:

(13) $$U(jw)=-\phi (jw)=\frac{\text{jwR}}{1+\text{jwCR}}Q(jw)$$

where *U* is the observational voltage, *ϕ* is the potential of the hemisphere probe. Based on Equations (9) and (13), one will have:

(14) $$\phi (jw)=-\frac{\text{jwR}}{1+jw(C+\lambda )R}Q_{1}(jw)$$

Gajewski [12] indicated that electrostatics induction depends strongly on the capacitance of the probe itself as well as of the whole system consisting of an electrostatic probe and a signal conditioner circuit. It must not be neglected to obtain actual theoretical results. Thus the function in the time domain is formulated in terms of convolution as Equation (15), where ⊗ is the convolution operator:

(15) $$\phi (t)=-\frac{1}{\left(C+\lambda \right)}e^{-\frac{t}{(C+\lambda )R}}⊗\frac{dQ_{1}(t)}{dt}$$

Supposing a unit point charge moves in the gas flow parallel to the pipeline with a velocity *v* and the original position is at (*x*_0_, *y*_0_, *z*_0_), then the input of the electrostatic sensor is considered as a unit impulse signal *δ*(*x* − (*x*_0_ + *vt*),*y* − *y*_0_, *z* − *z*_0_) along the ***x***-axis orientation. Thus the unit impulse response function of the probe is written as follows:

(16) $$h\left(t\right)=-Q_{1}(t)=\int \delta \left(x-(x_{0}+vt),y-y_{0},z-z_{0}\right)s\left(x,y,z\right)\text{dxdydz}=s(x_{0}+vt,y_{0},z_{0})$$

The output signal in the time domain can also be written in terms of convolution based on Equations (15) and (16):

(17) $$U=-\phi (t)=\frac{a\nu }{\left(C+\lambda \right)}e^{-\frac{t}{(C+\lambda )R}}⊗(x_{0}+\nu t){\left[{\left(x_{0}+\nu t\right)}^{2}+y_{0}^{2}+z_{0}^{2}\right]}^{-\frac{3}{2}}$$

The output voltage *U*′ for processing is amplified by the amplifier. It is assumed the amplifier gain is *k*, then one will have *U*′ = *kU*. Generally, *λ* is relatively smaller than the equivalent capacitance *C* since it is affected by the small dielectric permittivity of free space. In the considered approximation, one can use *C* instead of *C* + *λ* in Equation (17) to simplify numerical simulations.

## 3. Numerical Simulations

In this section, the theoretical model of the hemisphere-shaped sensor is numerically calculated and analyzed using the Matlab software. The spatial sensitivity, the homogeneity of the spatial sensitivity and the time-frequency response reflecting the performance of an electrostatic sensor are estimated respectively.

### 3.1. The Spatial Sensitivity of the Hemisphere-Shaped Electrostatic Sensor

**Figure 4.**
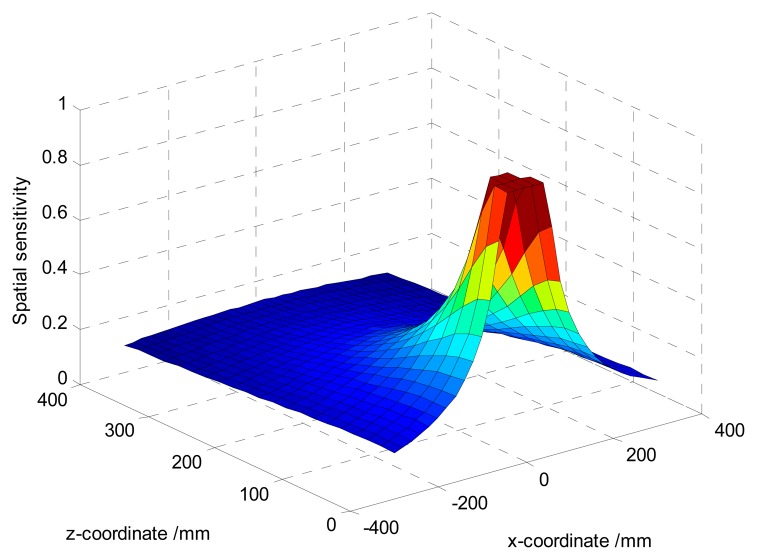
Variations of the spatial sensitivity with ***x*** and ***z***.

**Figure 5.**
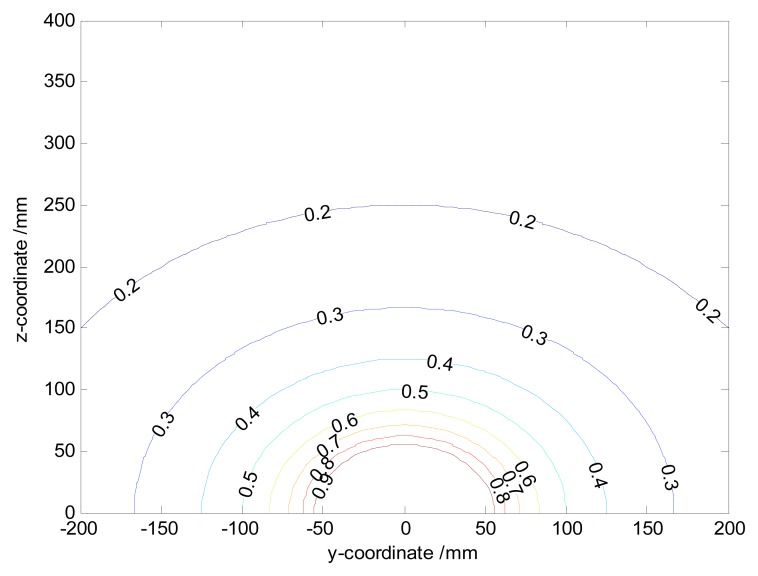
The contours of the spatial sensitivity.

The spatial sensitivity determines the magnitude of the output signal. Since the analytical function of the spatial sensitivity in Equation (11) is 3D-symmetrical and elementary, fixing any one of *x*-axis, *y*-axis or *z*-axis, the difference of simulated spatial sensitivity curves in the 2D-plane can be identified in terms of variable regions. For instance, if the radius of the pipeline is set as *L* = 200 mm, the various regions hold as follows: *x* ∈ [−∞, +∞], *y* ∈ [−*L*, + *L*], *z* ∈ [0, 2*L*] and $\sqrt{y^{2}+{\left(z-L\right)}^{2}}<L$. With the setting of *a* = 50 mm, *y*_0_ = 0 mm, the sensing plane of the hemisphere-shaped sensor is shown in Figure 4. It is obvious the spatial sensitivity increases with the decreases of | *x* |, | *z* |, so that one can infer that the spatial sensitivity increases with the decreases of | *x* | due to symmetry. The maximum value 1 is achieved at $\sqrt{x^{2}+z^{2}}=a$ when charges are near the hemispherical surface. It indicates the signals are mainly generated by particles near the probe. In other words, the spatial sensitivity is quite localized around the probe. Also one can find curves are truncated because charges can impossibly reach inside the hemisphere-shaped sensor. To further verify the 3D-symmetry of the spatial sensitivity, the contours of the spatial sensitivity on the cross-section of the pipeline with *x*_0_ = 0 mm are shown in Figure 5. It is evident the contours radiate as symmetrical rings.

**Figure 6.**
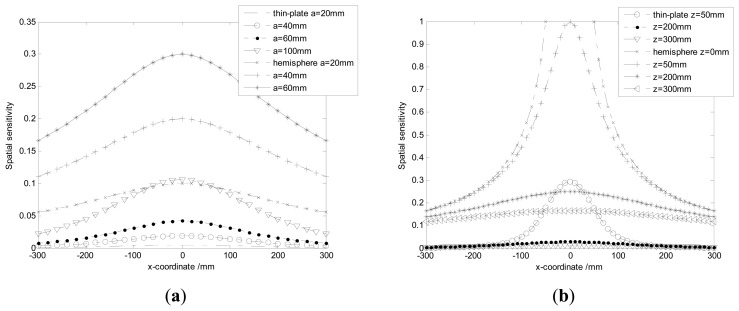
Variations of the spatial sensitivity with ***x*** for different radii (**a**) and ***z*** (**b**).

A circular thin-plate-shaped electrostatic sensor is taken as the representation of non-intrusive sensors, so that the spatial sensitivity is compared with the hemisphere-shaped sensor. When the initial settings are *y*_0_ = 0 mm, *z*_0_ = *L*, Figure 6a shows the variations of the spatial sensitivity with ***x*** for different radiuses of sensors. It is easy to find the spatial sensitivity of the hemisphere probe is proportional with its radius, which is consistent with the theoretical results in Equation (11). In practice, the radius of the probe is determined by the tradeoff between spatial sensitivity and the gas flow disturbance. Also the spatial sensitivity of the circular thin-plate-shaped sensor is much lower than the hemisphere-shaped sensor as shown in Figure 6a. Setting the radius as *a* = 50 mm, Figure 6b shows the variations of the spatial sensitivity with ***x*** for different ***z***. Obviously, the spatial sensitivity of the sensors increases with the decreases of | *z* |. It also indicates the similar compared conclusions as obtained from Figure 6a—the spatial sensitivity of the circular thin-plate-shaped sensor is much lower than the hemisphere-shaped sensor. In fact, even though *z* = 0 mm is set, the spatial sensitivity of the circular thin-plate-shaped electrostatic sensor cannot achieve a value of 1 due to its sensing characteristics.

### 3.2. The Homogeneity of the Spatial Sensitivity

**Figure 7.**
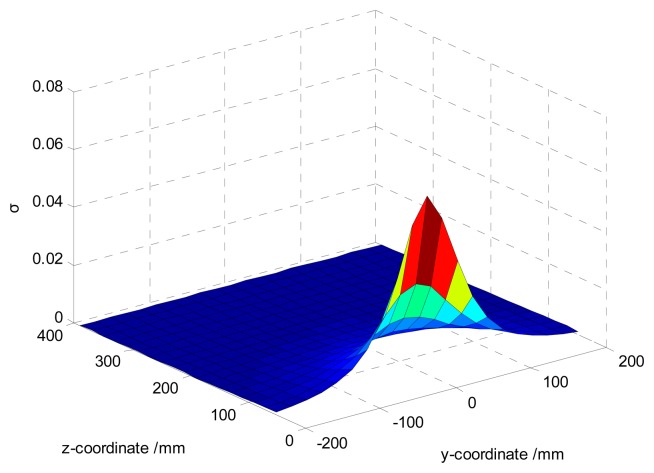
The homogeneity of the spatial sensitivity.

The homogeneity of the spatial sensitivity is another important performance parameter that determines sensing field. In this Section, the variance of the spatial sensitivity along the *x*-axis is defined to estimate the homogeneity of the spatial sensitivity due to symmetry. It is assumed that the related variables are limited as $\sqrt{y^{2}+z^{2}}>a$, *x* ∈ [−*N*, *N*], one will have:

(18) $$\sigma (s(y,z))=\frac{\int_{-N}^{N}{\left(s(x,y,z)-\bar{s}(y,z)\right)}^{2}dx}{2N}=\frac{a^{2}}{N}\frac{\arctan(N/\sqrt{y^{2}+z^{2}})}{\sqrt{y^{2}+z^{2}}}-\bar{s}{\left(y,z\right)}^{2}$$

where $\bar{s}(y,z)=\frac{\int_{-N}^{N}s(x,y,z)dx}{2N}=\frac{a}{N}\ln\frac{N+\sqrt{N^{2}+y^{2}+z^{2}}}{\sqrt{y^{2}+z^{2}}}$ is the mean value of the spatial sensitivity. It is easy to find $\underset{N\to \infty }{\lim}\sigma (s(y,z))=0$. Setting the parameters in Equation (18) as *N* = 300 mm, *z* ∈ [50 mm, 400 mm], the variations of the homogeneity of the spatial sensitivity with *y*, *z* are shown in Figure 7. One can find *σ* increases with the decreases of | *y* |, | *z* |. It indicates the hemisphere-shaped sensors have higher spatial sensitivity with less homogeneous near the hemispherical surface and *vice versa*.

**Figure 8.**
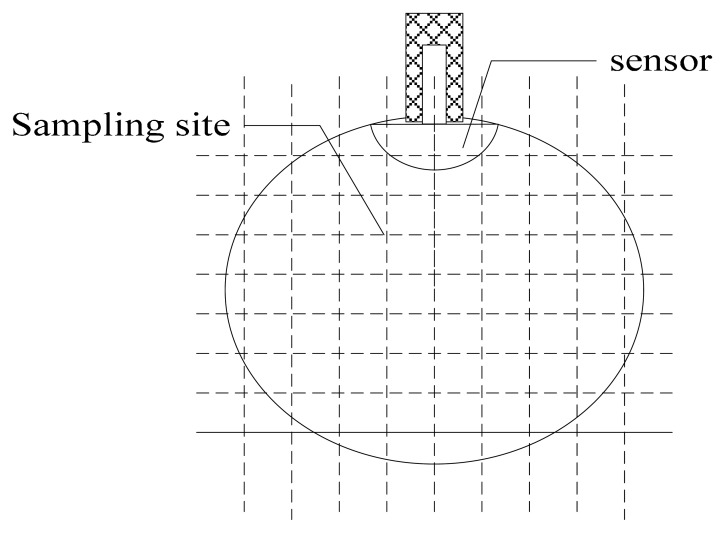
Sampling sites of the spatial sensitivity.

**Figure 9.**
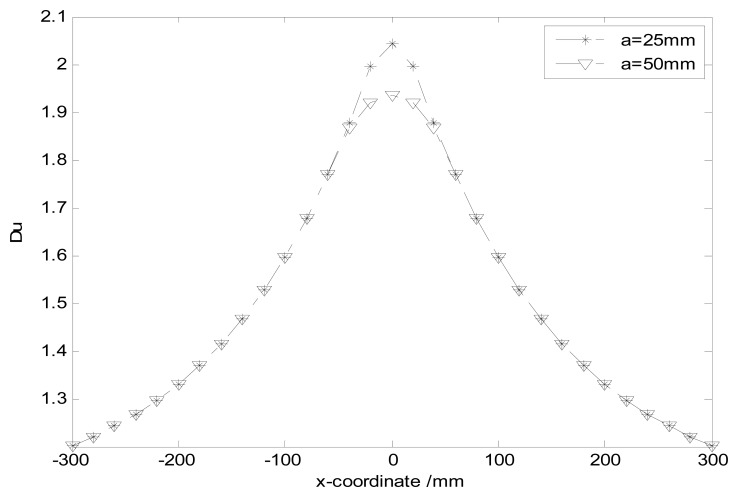
Variations of *Du* with ***x*** for different radii.

Within the pipeline, the particles always move past the probe with the gas flow. Hence improving the homogeneity and spatial sensitivity on the cross-section of the pipeline is more beneficial to detect particles and generate high output signals. Especially the homogeneity of the high sensitivity is frequently mentioned in practice. Since *σ* cannot reflect the related characteristic, another estimated coefficient is redefined. As shown in Figure 8, *Ns* sampling sites of the spatial sensitivity are built, whose value are denoted as *s*(*n*). Then the estimated coefficient can be expressed as:

(19) $$Du=\frac{\bar{S}h}{\bar{S}}=\frac{\bar{S}h}{\underset{N_{s}}{\text{∑}}s(n)/Ns}$$

where *S̄h* is the mean value of the first *N_s_*/4 sampling sites with the highest sensitivity. *S̄* is the mean value of the spatial sensitivity. The smaller *Du* indicates that the high sensitivity is more homogeneous. The variations of *Du* with *x* are shown in Figure 9.

One can find *Du* is almost the same for the different radii of the probe. It demonstrates the homogeneity of the high sensitivity has been little affected by the size of the probe. Incidentally, using a hemisphere-shaped sensor array instead of a single hemisphere-shaped sensor may be a good way to increase the homogeneity of the high sensitivity, and will be the topic of future work.

### 3.3. The Characteristic Analysis of the Output Signal

#### 3.3.1. The System Frequency Response of the Hemisphere-Shaped Sensor

The frequency response characteristics are critical parameters reflecting the performance of an electrostatic sensor, which can provide guidelines for optimal designing the hemisphere-shaped electrostatic sensor. The frequency response function of the hemisphere probe can be transformed from Equation (16):

(20) $$H(jw)=F(s(x_{0}+\nu t,y_{0},z_{0}))=\frac{e^{jw\frac{x_{0}}{v}}}{\nu }S(\frac{jw}{\nu })=\frac{2ae^{jw\frac{x_{0}}{v}}}{\nu }K_{0}(\sqrt{y_{0}^{2}+z_{0}^{2}}\left|\frac{w}{\nu }\right|)$$

where *K*(•) is the modified Bessel function of the second kind. Then the system frequency amplitude response is formulated as follows:

(21) $$\left|U(f)\right|=\frac{4\pi \text{aRf}}{\nu \sqrt{1+{\left(2\pi f(C+\lambda )R\right)}^{2}}}K_{0}(\sqrt{y_{0}^{2}+z_{0}^{2}}\left|\frac{2\pi f}{\nu }\right|)$$

**Figure 10.**
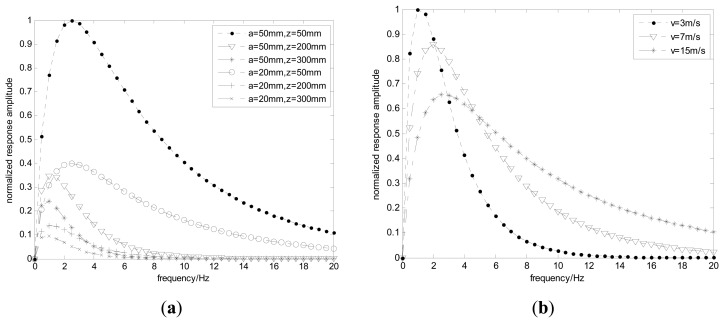
System frequency responses for different radii (**a**) and *v* (**b**).

Equation (21) indicates the system frequency amplitude strongly depends on the original position *y*_0_, *z*_0_, the velocity *v* of the charge and the radius of the probe. When the related parameters are set as *y*_0_ = 0, *v* = 3 m/s, *R* = 500 MΩ, *C* = 100 pF, Figure 10a simulates the system frequency responses for different *z*_0_ and probe radii. It can be seen that the system frequency response acts as a band-pass filter and the amplitude decreases with the decreases of the radius, but the bandwidth shows little changes. Also both the amplitude and bandwidth increase with the decreases of *z*_0_. Obviously, the input signal produced by the debris near the hemispherical surface should be more easily detected. In other words, the signal bandwidth is mainly determined by the particles near the probe.

The variations of system frequency responses with the velocity of the particle are shown in Figure 10b. Unlike the amplitude, the bandwidth becomes wider with the increases of the velocity. In practice, there are diverse types of particles with different characteristic frequency. Hence, checking maxima frequencies in the power spectrum of the output signal allows detecting different particles. Additionally, since the velocity of the particle depends on the gas flow, the bandwidth increases when the velocity of the gas flow increases.

**Figure 11.**
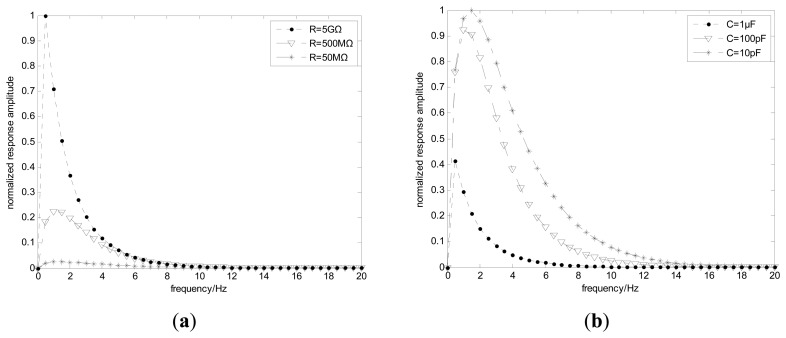
System frequency responses for different resistances (**a**) and capacitances (**b**).

With the settings of *y*_0_ = 0, *z*_0_ = 200 mm, *v* = 3 m/s, *C* = 100 pF the variations of system frequency responses for different resistances *R* are shown in Figure 11a. It can be seen the amplitude decreases with the decreases of *R*, but the bandwidth is almost unchanged. Figure 11b shows the variations of system frequency responses for different capacitances *C* when *R* = 500 MΩ is set. Unlike Figure 11a, both the amplitude and bandwidth have changed with the variation of *C*—the smaller *C* causes a frequency signal with larger amplitude and bandwidth. Hence the results indicate the larger *R* and smaller *C* of the circuit should be a good choice.

**Figure 12.**
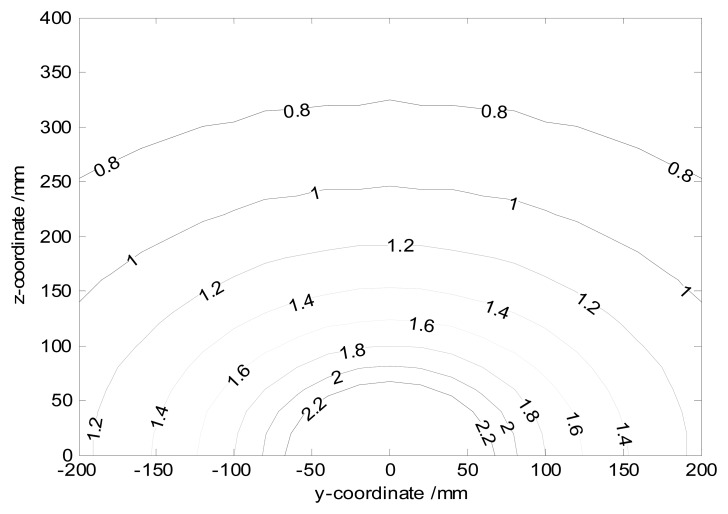
The peak spectral component.

As the previous discussion from Figure 10, farther charge streamlines from the probe induce signals with smaller frequency amplitude. Hence the flow distribution can also be inferred from the spectral density information. Figure 12 shows the peak spectral component of the signal that is induced by charges with different *y* and *z*. The corresponding parameters are set as *v* = 3 m/s, *R* = 500 MΩ, *C* = 100 pF. In practice, one can get the location of the real sensor signal from Figure 12. Identifying the real location of the concentrated bulk of the flow or debris in gas path is more possible.

#### 3.3.2. The System Time Response of the Hemisphere-Shaped Sensor

**Figure 13.**
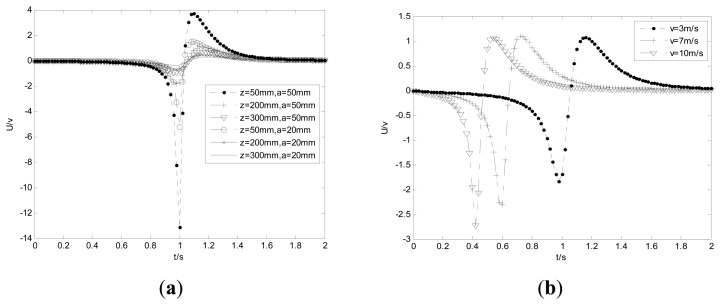
System time responses for different radii, *z*_0_ (**a**) and *v* (**b**).

In this section, the characteristics of the output signal with a unit impulse input signal are estimated. Supposing the original position of the particle is at (−3, 0, 0.2) m and the velocity is set as *v* = 3 m/s, one can find the output voltage increases with the increases of the radius in Figure 13a. Besides, the output voltage includes two impulsive signals, because it is the derivative of the induced charge. Furthermore, the smaller the distance between the charge and the probe, the larger the voltage amplitude. Figure 13b illustrates that the larger velocity of the particle causes a voltage with a larger amplitude. Obviously, the response time is also shorter with the larger velocity.

**Figure 14.**
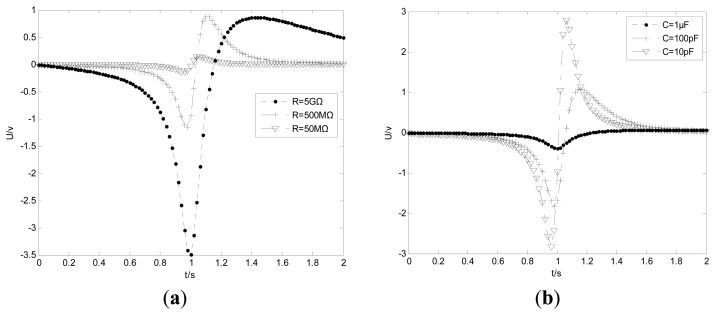
System time responses for different resistances (**a**) and capacitances (**b**).

One can see that the amplitude of the negative impulsive signal is larger than the positive one, which is different from many articles [6,8,16]. *C* and *λ* in Equation (17) are the main reasons with the responsibility for the attenuation of the voltage amplitude, which cannot be neglected. In fact, the time constant *τ* = *R*(*C* + *λ* ) is the delay factor. Figure 14 shows the system time responses for different resistances and capacitances. It can be seen from Figure 14a that when the capacitance is set as *C* = 100 pF, both the voltage amplitude and delay increases with the increases of the resistance. In contrast, using the setting of *R* = 500 MΩ, the voltage amplitude decreases with the increases of capacitance, while the delay still increases. Thereby one can infer that the delay increases with the increases of *τ* from the common results. Additionally, to decrease the attenuation of the voltage amplitude, one should choose smaller capacitance and larger resistance, consistent with the previous conclusions.

In the previous simulations, one supposes that particles move along the pipeline with constant velocity. If particles start to move at the position (*x*_0_, *y*_0_, *z*_0_) with gravitational acceleration, the output signal can be expressed as follows:

(22) $$U=\frac{\text{aqg}}{\left(C+\lambda \right)}\int_{0}^{t}e^{-\frac{t-\tau }{(C+\lambda )R}}\tau (x_{0}+\frac{1}{2}g\tau^{2}){\left[{\left(x_{0}+\frac{1}{2}g\tau^{2}\right)}^{2}+y_{0}^{2}+z_{0}^{2}\right]}^{-\frac{3}{2}}d\tau$$

**Figure 15.**
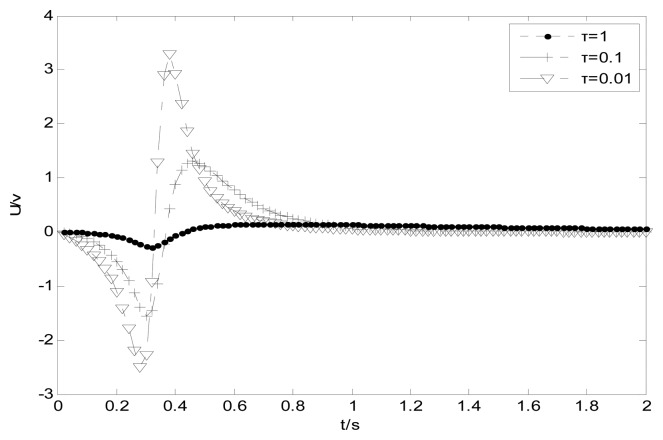
System time responses with gravitational acceleration.

The simulation results are shown in Figure 15.

The attenuation of the voltage amplitude caused by the time constant *τ* is verified again. Otherwise, the positive impulsive signal should be larger than the negative one as the modeled curve shown with *τ* = 0.01 because of the increases of the velocity.

## 4. Conclusions

The electrostatic probe is the key component for capturing the changes of total charges in the gas path or measuring particle concentration and size in a gas-solid flow. Due to their advantages of low cost and robustness, various probes have been widely used in industrial applications. Non-intrusive probes usually have little spatial sensitivity and weak output signals as the result of the pipeline shields or their small induced area. Conversely, the intrusive probes have the opposite advantages while they often affect the gas flow. The common disadvantages of those sensors are either that their electrodynamic models cannot be resolved due to their complicated shapes, *i.e.*, rod-shaped, or the obtained analytical functions are too complicated to study their sensing characteristics. Unlike those sensors, a novel hemisphere-shaped sensor was investigated in this paper, and the corresponding analytically 3D-symmetrical model was obtained in terms of elementary functions, which is an important advantage in many applications.

In this paper, the Green function of the point charge outside the hemisphere probe was derived first. Then the spatial sensitivity and time-frequency response functions were also directly formulated based on the Green function. The numeric simulations estimated the performance of sensors in terms of the spatial sensitivity, the homogeneity of the spatial sensitivity and the time-frequency response. The main results can be summarized as follows.

(1) The spatial sensitivity of the hemisphere-shaped sensor is 3D-symmetrical and elementary, which is much higher than that of a non-intrusive sensor.
(2) Hemisphere-shaped sensors have a highly inhomogeneous spatial sensitivity near the hemispherical surface and *vice versa*.
(3) The temporal frequency response acts as a band-pass filter. The time-frequency characteristics are determined by the spatial position and velocity of the charged particle, and the radius of the probe, as well as the equivalent resistance and capacitance of the circuit.
